# Differences in health visiting contacts for children of mothers with and without a history of adversity-related hospital admissions in England (2018/19 to 2019/20): an analysis of novel linked community health and hospital data.

**DOI:** 10.23889/ijpds.v9i5.2873

**Published:** 2024-09-18

**Authors:** Louise Mc Grath-Lone, Jenny Woodman, Amanda Clery, Mengyun Liu, Catherine Bunting, Katie Harron

**Affiliations:** 1 University College London

## Objective

Explore differences in children’s contact with health visiting (HV) services by pre-birth exposure to maternal adversity.

## Approach

For the first time, we linked the Community Services Dataset (CSDS), which records HV contacts for children in England from 1 April 2018 to 31 March 2020, to Hospital Episode Statistics (HES) which contains births and mothers’ hospital admissions in the preceding 3 years. We used admissions that included ICD-10 codes related to mental health, substance use and violence to identify children born to mothers with a history of adversity. We compared characteristics of children’s HV contacts (i.e. number, mode, location, duration) stratified by maternal adversity.

## Results

Overall, 85.2% of 626,478 children in CSDS linked to a birth record in HES. Among children receiving HV contacts, 11.7% were exposed to maternal adversity: 11.1% mental health, 1.3% substance use, 0.4% violence. Children with maternal adversity had more HV contacts than other children (mean = 3.8 vs 2.7, p<0.001). There were no differences in contact mode (i.e. face-to-face or phone) for children with maternal adversity compared to others, but their contacts were longer (44.5% lasting <30 minutes vs 35.1%, p<0.001) and more likely to be at home (56.3% vs 47.8%, p<0.001).

## Conclusions

HV services respond in different ways to children with and without pre-birth exposure to serious maternal adversity, reflecting the “proportionate universalism” that underpins HV services in England.

## Implications

The differential service response for vulnerable children may require additional resources (e.g. staff, training) which should be considered when designing and funding HV services.

